# Treatment of Inflammatory Rheumatism

**Published:** 1854-03

**Authors:** O. C. Gibbs

**Affiliations:** Perry, Ohio


### *On the Treatment of Inflammatory Rheumatism.*

BY O. C. GIBBS, M. D., PERRY, OHIO.

The present is, emphatically, the age of improvement in therapeutic science. Yet, in the enthusiasm for things which are new, our profession is, perhaps, too apt to adopt newly suggested remedial means, frequently to the neglect of those honored by time and the support of worthy names. This is certainly more laudable than the opposite extreme, that of clinging to the doctrines and practices in which we were first educated, regardless of the pathological or therapeutic improvements of the age, but, nevertheless, is reprehensible, if lacking due consideration in reference to comparative merit.

New theories in regard to the nature, and new plans in reference to the treatment of inflammatory rheumatism, have followed each other in rapid succession, and been supported by a nearly equal array of names. In reference to remedies, blood letting, purgatives, calomel, tartar-emetic, opium, nitre, colchicum, guaiac, quinia, phosphate of ammonia, carbonate of potassa and soda, and lastly, lemon-juice, have each had their advocates.

In view of this discrepancy of therapeutic opinion, Prof. Wood, of Philadelphia, whose opinions, in our humble judgment, are always worthy of consideration, advises blood letting, purgatives, and refrigerant diaphoretics. If the disease is not subdued in *two weeks*, calomel with opium is advised; and, in adynamic conditions of the system, quinia. In our limited experience with this disease, we have tried several plans of treatment, and, so far, have found none worthy of comparison with that first suggested by Dr. Chambers and published by Dr. Hope, nearly twenty years ago. The plan consists as our readers are doubtless well aware, in venesection, if necessary, followed by eight or ten grains of calomel and one and a half grains of opium, for an adult, at bed-time; and, in the morning, by a strong black dose, sufficient to secure four or five stools. This night and morning medication, is to be continued until a cure is effected, excepting the calomel, which is to be discontinued when the symptoms considerably abate, or the gums become at all tender. With this treatment is con. joined thrice a day, a saline draught, containing five grains of Dover's powder, and fifteen or twenty minims of wine of colchicum.

Under this treatment, early commenced, we have never seen endo- or peri-cardiac complication, or known a case to protract more than ten days. Perhaps, from our limited observation, we have placed too high an estimate upon the success of this plan, and it is only because we think some modern authors have estimated it too low, that we make these remarks. It is due to Dr. Owen Reese, to say that lemon-juice has never been tried by us, as suggested by him.

From our cases we select the two following, illustrative of this plan of treatment.

CASE 1st.—February 1st, 1850, was called to see Jane T——, aged five years; had been ill one day; the left shoulder was considerably swollen, very red and painful; the right shoulder was slightly affected. Her father died from some cardiac disease: had previously been much afflicted with rheumatism, and, for the last few years of life, almost helplessly so. Three grains of calomel and five of Dover's powder were ordered at bed-time, to be followed, in the morning, by a sufficient black dose to secure a free cathartic action; a powder composed of three grains each of powdered colchicum and Dover's powder, was ordered to be taken two hours after the black dose, to be repeated every four hours through the day. This treatment was continued until the third day, when the calomel was omitted, and the powders ordered every six hours instead of four. On the fifth day the patient was discharged cured.

CASE 2d.—December 4th, 1851, was called to see Mr. E——, aged forty-five years, had been troubled with rheumatism for several weeks, and had been treated by a Thompsonian with colchicum, Dover's powder, &c. The knees and ankles had been the seat of disease, affecting each limb singly and alternately. When first seen by us, the left knee and ankle were considerably swollen and painful, with but little redness and fever; the patient was not confined to the house, though unable to attend to business. The disease was evidently subacute or chronic, and the plan of treatment proposed by Dr. Budd, in the London Lancet, was adopted. The following was advised: five grains of bicarbonate of potassa and two grains of iodide of potassium, dissolved in an ounce of water, three times a day, and five grains extract colocynth at bed-time; with alkaline lotions to the affected joints. A milk diet, and a daily warm bath were also enjoined. This treatment was continued for two weeks without benefit.

Thirty grains of acetate of potash, in camphor mixture, every four hours through the day; four grains of quinia, in a pill, three times a day; and ten grains of Dover's powder at bed-time, the bowels to be opened with calomel and colocynth, when necessary, were now ordered, as recommended by Dr. Golding Bird, of London. This treatment was continued for two weeks, also without benefit. The plan suggested by Dr. Chambers, as given above, was now adopted, excepting the venesection. Eight grains of calomel and one of opium, was given every night, followed by black dose in the morning, and twenty drops of wine of colchicum with five grains of Dover's powder, in saline draught, three times a day. The patient rapidly convalesced, and was discharged, cured, on the seventh day from the commencement of this treatment.

Dr. Watson says he never saw but one case of inflammatory rheumatism, occurring before puberty, uncomplicated with cardiac disease. The first case given here, though the patient was quite young, was entirely exempt from such complications.—*Med. Examiner.*

